# Transformation of industrial wastewater into copper–nickel nanowire composites: straightforward recycling of heavy metals to obtain products of high added value

**DOI:** 10.1038/s41598-020-76374-x

**Published:** 2020-11-05

**Authors:** Tomasz Wasiak, Pyry-Mikko Hannula, Mari Lundström, Dawid Janas

**Affiliations:** 1 Department of Chemistry, Silesian University of Technology, B. Krzywoustego 4, 44-100 Gliwice, Poland; 2 Department of Chemical and Metallurgical Engineering, School of Chemical Engineering, Aalto University, Vuorimiehentie 2, 02150 Espoo, Finland

**Keywords:** Materials science, Nanoscale materials, Nanowires

## Abstract

Large amounts of industrial metal containing process and waste solutions are a growing issue. In this work, we demonstrated that they could be transformed into materials of high added values such as copper-nickel nanowires (CuNi NWs) by simple chemical reduction. A thorough investigation of the parameter space was conducted. The microstructure of the obtained material was found tunable depending on the employed concentration of precursor, reducing agent, capping agent, pH, temperature, and reaction time. Moreover, the obtained product had a strong magnetic character, which enabled us to separate it from the reaction medium with ease. The results open new perspectives for materials science by proposing a new type of nanostructure: composite NWs of very promising properties, with metallic elements originating directly from industrial process solution.

## Introduction

Handling of industrial waste and process solutions is a growing issue that requires new routines, but can also provide new business opportunities. In particular, acidic wastewater from industrial plants containing heavy metals, such as nickel, copper, and arsenic, is one of the main undesirable by-products which have to be dealt with. At the same time, the ever-increasing global demand for metals for electronics^1^ and other growing fields enlarges the need for metallurgical industry operations for metals, such as Co, Ni, and Cu. As such, efficient removal and re-use of metals from the various processes and wastewaters is necessary for the sustainable growth of these industries. This approach has a dual purpose. Firstly, some of these species are toxic even at low concentrations, which endangers flora and fauna^2–6^, when such waste solutions end up in the environment. Secondly, these resources are often of high commercial value; therefore, having a way to recover them is of the economic essence. Numerous treatment techniques are used to obtain valuable materials from wastewater such as precipitation^7^, adsorption^8^, cementation^9^, membrane filtration^10^, and electrochemical recovery^11,12^. Despite their large share in the purification of industrial wastewaters, they have limitations like maximum uptake of a specific metal or high equipment cost.

Simultaneously, the promising nature of nanomaterials^13,14^ encourages scientists to search for new paths to obtain them feasibly and at a lower cost. At present, the high price is still one of the critical constraints limiting the use of nanomaterials in real life. Recently it has been observed that green synthesis of carbon-based nanomaterials from biomass may result in a significant decrease in the price of the end product since such precursors are inexpensive and easily accessible^15–17^. In particular heavy metals have a large share in process and wastewaters, so it would be reasonable to explore if they can also serve as a precursor similarly. Having a method to convert them to high added value products like nanomaterials would open a spectrum of applications because the cost barriers to enter the market would be partially reduced. Nanomaterials are thought to be crucial for the further development of electronic devices due to their unique electrical, mechanical and thermal properties^18–21^. Nanowires (NWs) are an example of such species, which are defined as nanostructures with diameters less than 100 nm, the length of which is orders of magnitude larger than their diameter. There are many different techniques to obtain NWs such as Chemical Vapour Deposition (CVD)^22^, Plasma Enhanced Chemical Vapour Deposition (PECVD)^23^, or laser ablation^24^. What is more, vapour–liquid–solid (VLS) methods using specific substrates coated with a catalyst such as gold^25^ are also known to provide such nanostructures. The key advantage of these techniques is that the diameter of the resulting product can be somewhat controlled at the expense of the high cost of the equipment or the process itself^26^. On the other hand, solution-based synthetic techniques receive a lot of attention nowadays due to lower costs of equipment and the ability to scale up the production in a straightforward way. An example of such a process, which has gained the special focus of the community, is the polyol synthesis through which silver nanowires (Ag NWs) can be obtained. A simple modification of the synthesis parameters such as the concentration of precursor, capping agent, or reaction temperature allows for the controlled production of nanostructures with different morphologies^27,28^. Despite the favorable properties of Ag NWs, which can be used as transparent conductive coatings, CuNWs are in parallel getting a large share of attention due to similar electrical conductivity at a much lower cost^29^. As a consequence, at present, CuNW fabrication by reduction of copper ions from simple synthetic solutions with high purity and no other metallic species is a well-studied method in the literature^30–32^. Different reactants and process parameters have been studied to achieve NWs with a high aspect ratio and appreciable electrical conductivity. Typically CuCl, Cu(NO_3_)_2,_ or Cu(acac)_2_ are used as a precursor and hydrazine as a reducing agent. While the properties of the obtained materials are encouraging, application of synthetic solutions to produce them makes the process, and thus the product, expensive.

To the best of our knowledge, there have not been any attempts to synthesize Cu NWs from complex chemical environments containing multiple metallic elements such as metallurgical processes or waste solutions. Possibility for the manufacture of nanomaterials from an impure solution containing other chemical species than just copper would have a range of advantages. First and foremost, the presence of metals such as cobalt or nickel may offer the possibility of synthesis for a high added-value composite nanomaterial with desirable properties such as ferromagnetism. Secondly, such solutions can be acquired at low cost in high quantities, so the nanostructures would be cheaper to produce. Last but not least, such a technique would be a useful way to utilize existing processes and wastewater solutions directly to minimize our impact on the environment.

In this work, we show the technology of preparation of CuNi NWs from industrial process solutions for the first time. No pre-processing of the feed was required besides dilution with water to reach an appropriate concentration of metal ions. By exploring the parameter space of the synthesis procedure, such as time, temperature, or concentration of the solution and employed chemical agents, we were able to tune the microstructure of the resulting CuNi NWs. The product obtained under optimized conditions was long, thin, and magnetically active due to the successful implantation of Ni. This property enabled us to separate it with ease from a complex precursor solution containing 14 metallic elements. The resulting material was of a highly uniform structure and contained only Ni and Cu.

## Experimental

The industrial solution used as a precursor solution for NW production originated from copper electrorefining (ER). It was composed predominantly of copper and nickel with high sulfate concentration and high acidity. Characterization of its chemical composition was done by ICP-OES (Inductively Coupled Plasma—Optical Emission Spectrometry; Perkin Elmer Optima 7100 DV, USA), and it is given below in Table 1.

**Table 1:** Composition of the source material.

	Original Cu process solution (pH 1) (mg/L)	After dilution to 0.12 M Cu^2+^ (pH 1) (mg/L)	After dilution to 0.06 M Cu^2+^ (pH 1) (mg/L)	After dilution to 0.03 M Cu^2+^ (pH 1) (mg/L)
Cu	44,000.00	7620.00	3810.00	1905.00
Ni	19,300.00	3342.41	1671.20	835.60
As	23,300.00	4035.14	2017.57	1008.78
Sb	196.00	33.94	16.97	8.49
Bi	220.00	38.10	19.05	9.53
Fe	165.00	28.62	14.31	7.16
Al	20.00	3.46	1.73	0.87
Ag	< 5.00	< 0.87	< 0.43	< 0.22
Au	< 5.00	< 0.87	< 0.43	< 0.22
Pt	< 5.00	< 0.87	< 0.43	< 0.22
Pd	< 5.00	< 0.87	< 0.43	< 0.22
Te	< 10.00	< 1.73	< 0.87	< 0.43
Se	< 10.00	< 1.73	< 0.87	< 0.43
Co	< 10.00	< 1.73	< 0.87	< 0.43
S	80,300.00	13,906.50	6953.25	3476.63

The additional chemical compounds used in this work were: Ethylenediamine (Merck, Finland), Hydrazine hydrate (55% aqueous solution, Merck, Finland), and Sodium hydroxide (Merck, Finland). All of them were of analytical grade. Deionized water was used for the solubilization of solid components or dilution of aqueous solutions to reach the appropriate concentrations.

**Figure 1:**
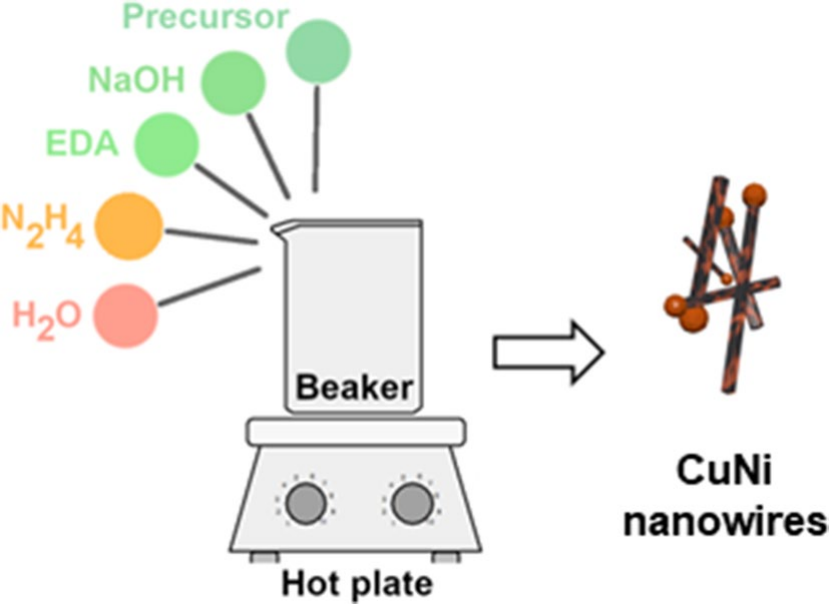
Scheme of the synthesis procedure.

For the synthesis of nanomaterials, we used parameter space similar to the one published by Chang and co-workers for Cu NW synthesis from a simple precursor^30^. First, the industrial process solution was diluted with deionized water to obtain the concentration of Cu^2+^ ions of 0.12 M, 0.06 M, or 0.03 M. After dilution of the process solution, the concentration of metallic species was in the range of what is typical for the industrial wastewaters. Then, 80 mL of NaOH (8–12 M) and 4 mL of the diluted precursor solution were combined in a beaker. The solution was then stirred with a magnetic stirrer while heating over a hot plate until reaching the selected temperature within the 60–85 °C range. Next, ethylenediamine (EDA, 0.1–0.3 mL) and hydrazine (N_2_H_4_, 0.05–0.2 mL) were added. The blue solution turned white, and the formation of a red precipitate was observed, which changed color to black after 2 min. The reaction was then conducted for another 10 or 60 min as indicated. The resulting precipitated product was separated and washed with deionized water five times to remove the adulterants. A magnet was used to facilitate the purification, which ensured that only magnetic CuNi NWs are harvested. The product was then dried at 100 °C in air for ca. 2 h until reaching stable mass. The scheme of the process is given in Fig. 1.

The synthesis procedure was investigated as a function of the concentration of precursor, the concentration of NaOH as well as by varying the volume of EDA and hydrazine. Moreover, we tested the influence of reaction time and temperature. All the combinations of the parameters employed for the reactions are summarized below (Table 2).

**Table 2:** Parameters employed for syntheses of CuNi NWs.

Sample	*C*_Precursor_ (Cu^2+^) (M)	*C*_NaOH_ (M)	*V*_*E*DA_ (mL)	*V*_Hydrazine_ (mL)	*T* (^o^C)	*t* (min)
*Starting parameters*						
S1	0.06	10	0.2	0.1	60	10
*Modified parameters*						
S2	0.03	10	0.2	0.1	60	10
S3	0.06	8	0.2	0.1	60	10
S4	0.06	10	0.1	0.1	60	10
S5	0.06	10	0.1	0.1	70	10
S6	0.06	10	0.2	0.05	60	10
S7	0.06	10	0.2	0.05	70	10
S8	0.06	10	0.2	0.1	70	10
S9	0.06	10	0.2	0.1	80	10
S10	0.06	10	0.2	0.1	85	60
S11	0.06	10	0.2	0.2	60	10
S12	0.06	10	0.2	0.2	70	10
S13	0.06	10	0.3	0.1	60	10
S14	0.06	10	0.3	0.1	70	10
S15	0.06	10	0.3	0.1	70	60
S16	0.06	12	0.2	0.1	60	10
S17	0.06	12	0.2	0.1	70	10
S18	0.06	12	0.2	0.1	70	60
S19	0.06	12	0.2	0.2	60	10
S20	0.06	12	0.3	0.1	60	10
S21	0.06	12	0.3	0.1	70	10
S22	0.06	12	0.3	0.1	70	60
S23	0.12	10	0.2	0.1	60	10

The microstructure of the obtained nanomaterials was characterized by a Scanning Electron Microscope (SEM, JEOL JSM-7500FA) with an acceleration voltage of 5 kV.

Energy-dispersive X-ray spectroscopy (EDX, JEOL JED-2300 Analysis Station) attached to the SEM was used for the characterization of the chemical composition of the materials. For selected samples, elemental mapping was performed. Carbon was excluded from the analysis since samples were mounted on the microscope stage with carbon tape.

## Results

### Synthesis of CuNi NWs under regular conditions

In our solution containing multiple elements, we expect co-precipitation of Cu and Ni according to the following redox reactions:

1 $$ 2{\text{Cu}}^{2 + } + {\text{N}}_{2} {\text{H}}_{4} + 4{\text{OH}}^{-} \to 2{\text{Cu }} + {\text{N}}_{2} + \, 4{\text{H}}_{2} {\text{O}} $$

2 $$ 2{\text{Ni}}^{2 + } + {\text{N}}_{2} {\text{H}}_{4} + 4{\text{OH}}^{-} \to 2{\text{Ni}} + {\text{N}}_{2} + 4{\text{H}}_{2} {\text{O}} $$

**Figure 2:**
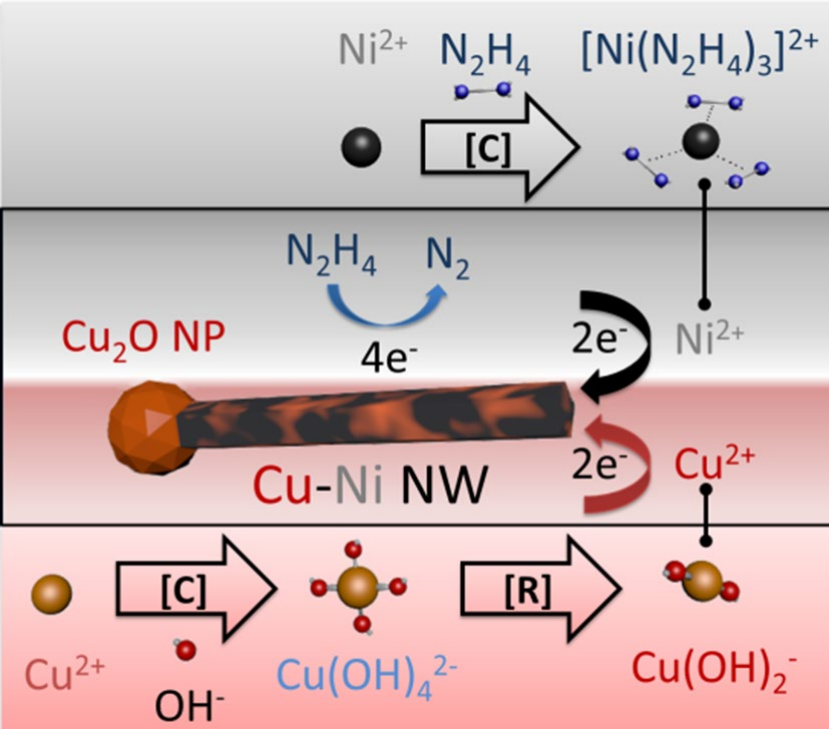
Growth of a CuNi NW. *C* complexation, *R* reduction.

In the beginning, the addition of Cu^2+^ ions to a solution with very high pH results in the formation of blue Cu(OH)_4_^2−^ complex. When hydrazine is introduced, the solution turns colorless due to complex transformation into Cu(OH)_2_^−^. Further reduction causes the nucleation of Cu_2_O nanoparticles, which act as seeds for NW growth in {110} plane^33,34^. Due to a higher concentration of Cu ions in the solution (Table 1), precipitation of Cu_2_O nanoparticles is favored kinetically as compared with the deposition of nickel oxides. At the same time, the solution also contains Ni^2+^ ions, which remain as Ni(OH)_2_. Once the Cu_2_O seeds are formed, reduction of Ni(OH)_2_ and Cu(OH)_2_^−^ causes Ni and Cu precipitation, respectively, on the surface of the seeds. Complexes formed during the reaction prevent the co-precipitation of the product with arsenic present in a relatively high amount. During the synthesis procedure, EDA acts as a capping agent and adsorbs on the sidewalls of the formed nanostructures, blocking the deposition of metal ions, which results in anisotropic growth^30,35,36^. This prevents the nanostructure from the preferential formation of spherical nanoparticles, which have a lower energy level. The most desirable properties of the product include long, straight, and thin NWs preferentially with the negligible presence of unwanted chemical species. The overall scheme of the synthesis mechanisms is shown in Fig. 2.

**Figure 3:**
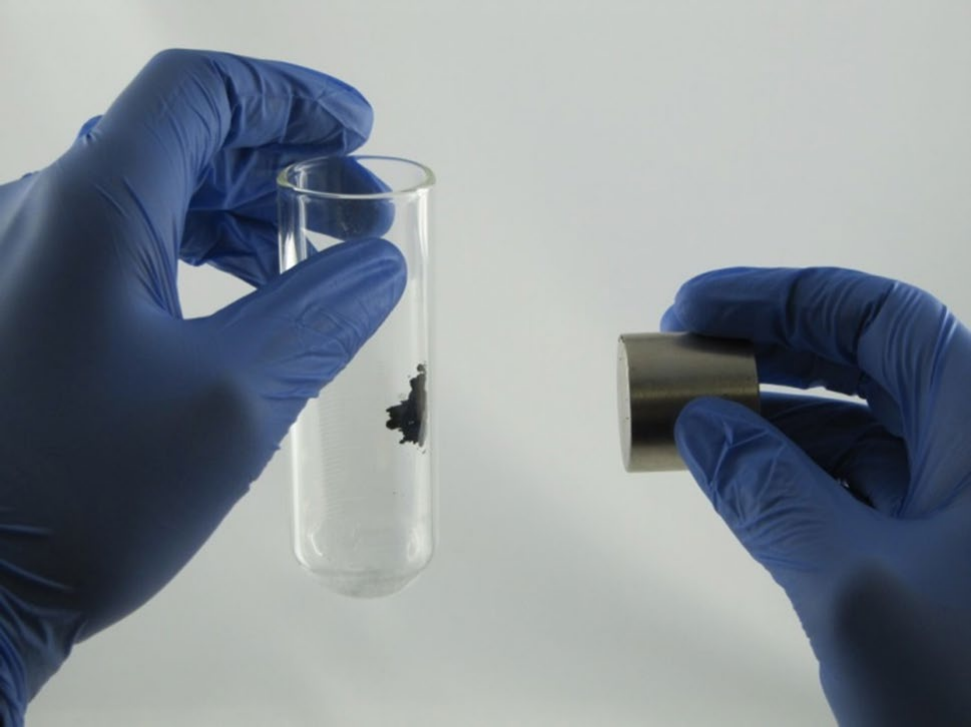
Magnetic character of CuNi NWs.

Successful incorporation of metallic nickel along with copper was also indicated with a strongly magnetic character of all the samples obtained (Fig. 3).

Initially, the reaction conditions for the synthesis were selected based on the literature^30^—referred to as S1 (0.06 M Cu^2+^; 10 M NaOH; 0.1 mL N_2_H_4_; 0.2 mL EDA; 60 °C reaction time 10 min). Then, the concentration of particular species crucial for the synthesis of NWs, time of the reaction, and the temperature of the process was modified (experiments S2-S23) to see their influence on the morphology and chemical composition.

**Figure 4:**
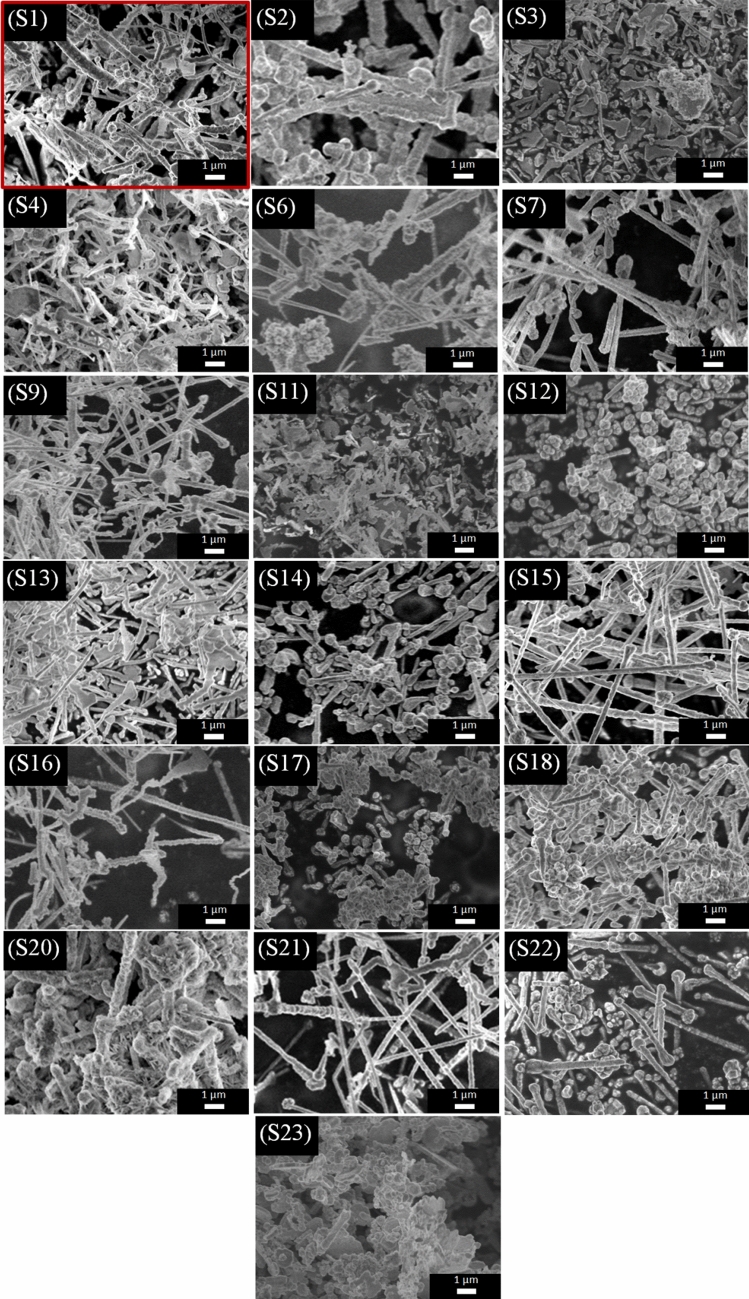
SEM micrographs of the obtained selected nanostructures. SEM micrograph of the first sample produced using reference conditions is highlighted (denoted as S1 in the text).

The product obtained from the first experiment S1 showed irregular and twisted NW morphology (S1, Fig. 4). When the temperature of the process was increased by 10 °C, straight and almost uniform NWs were obtained at 70 °C (S7, Fig. 4). However, a further increase in temperature up to 80 °C under these conditions (S9, Fig. 4) led to the synthesis of aggregated nanoparticles. Increasing the rate of Cu_2_O formation beyond a certain threshold caused a rapid decrease in the concentration of Cu^2+^, which reduced the driving force for anisotropic growth. It was evident that the kinetics of the synthesis reaction need to be appropriately controlled to create long and straight NWs.

### The influence of the concentration of the precursor solution

The metallurgical process solution of different concentrations of 0.03 M, 0.06 M, 0.12 M of Cu metal ions was employed (the concentration was adjusted by dilution with distilled water). When the concentration of metal ions was high (0.12 M), the main products were nanoparticles with a rough surface (S23, Fig. 4), with only a few nanorods discernible in the provided micrograph. It can be concluded that at a high concentration of precursor species, radial growth is favored rather than the formation of desirable 1D nanomaterials, i.e. NWs. At lower concentrations (0.03 M and 0.06 M), however, the syntheses gave mostly NWs and nanorods, which are of much lower aspect ratio (S1 and S2, Fig. 4). In these cases, the optimum amount of Cu and Ni ions in the solution enabled their anisotropic addition in a controllable fashion along the NW axis.

### The influence of concentration of the reducing agent

As a reductant, hydrazine was used in volumes of 0.05 mL, 0.1 mL, and 0.2 mL. Without varying other parameters, the best results were obtained for 0.1 mL (S1, Fig. 4). A higher amount of hydrazine 0.2 mL (S11, Fig. 4) led to disorganized precipitation and irregular shapes of obtained nanomaterials. A large number of short and thin proto-NWs could be detected in the micrograph. It can be concluded that an excessive amount of hydrazine beyond 0.1 mL hampered the NW growth both in the radial and longitudinal directions. Under highly reducing conditions, the formation of seed particles was preferred, which depleted the available amount of Cu and Ni for NW elongation. On the other hand, using 0.05 mL of hydrazine gave mostly NWs and nanorods, along with some nanoparticles (S6, Fig. 4). As expected from the literature^30,36^, a low concentration of hydrazine favors 1D nanostructures. However, it should be noted that these NWs were coated with unwanted sphere-shaped nanoparticles. Because the synthesis course is also temperature-dependent, we wanted to find out whether a change of this parameter would improve the microstructure. In combination with an elevated temperature of 70 °C, 0.05 mL of hydrazine gave better morphology of the nanomaterial due to the removal of the nanoparticle-like coating from NWs (S7, Fig. 4). This finding encouraged us to investigate the influence of the process temperature at a high concentration of hydrazine of 0.2 mL. Interestingly, it was found that increasing the temperature up to 70 °C under these process parameters gave aggregated material composed mostly of spherical nanoparticles (S12, Fig. 4). Similar to previous results (S11, Fig. 4), the reduction of precursor ions with high hydrazine concentration to form NW seeds was too fast to afford preferred anisotropic growth. An increase in temperature, in this case, resulted in even more pronounced radial growth of nanoparticles and NWs, which were notably larger as compared with the material produced at a lower temperature.

### The influence of concentration of the capping agent

To promote anisotropic growth, EDA is required as a capping agent, which blocks the addition of metal atoms to the sides of NWs^34^. Different amounts of EDA were examined (0.1 mL, 0.2 mL, and 0.3 mL). The synthesis reaction was found to be very sensitive to the amount of EDA in the solution. When 0.3 mL was used, longer NWs were obtained (S13, Fig. 4) as compared with the sample produced using starting conditions of 0.2 mL EDA (S1, Fig. 4). On the other hand, a lower volume of EDA (0.1 mL) was insufficient, which resulted in the formation of irregular nanoparticles and short NWs (S1, S4, Fig. 4).

The synthesis was also conducted at an elevated temperature of 70 °C for low (S5, Fig. 4) and high concentration (S14, Fig. 4) of EDA while keeping other parameters intact to probe the reaction mechanism. Similarly to the case when the concentration of hydrazine was excessive (S12, Fig. 4), obtained NWs were short and very much contaminated with spherical nanoparticles when 0.1 mL of EDA was employed. However, when both the temperature and the EDA volume were increased to 70 °C and 0.3 mL, respectively, the product was mainly composed of NWs with small diameters of moderate length. At this point, we decided to find out whether the increase of the reaction time would lead to elongation of the NWs. The results showed that, indeed, under appropriate conditions, prolonged time of the reaction is advantageous. Extension of the reaction time to 60 min promoted further growth of NWs, which eventually reached more than 10 µm (S15, Fig. 4). This means that a significant part of precursor ions remained unreacted after 10 min when the experiment was typically completed. In the micrographs of the elongated NWs, we discerned the characteristic spherical shapes at the NW ends, which are Cu_2_O seeds necessary for the growth as reported earlier by Rathmell et al.^34^.

### The influence of pH

Another investigated parameter was the concentration of NaOH. Since the reaction requires a strongly alkaline environment, it can be expected that increasing the amount of NaOH will improve the morphology of the product by the more favored formation of necessary metal ion complexes to drive the process. Experiments with a stronger NaOH solution (12 M) were conducted to confirm this hypothesis. As a result, NWs with small diameters mainly occurred in the product (S16, Fig. 4) at 60 °C. However, when the temperature was increased to 70 °C, only small nanoparticles with diverse morphology were produced (S17, Fig. 4). Promoted formation of complexes, due to excessive amount of OH^−^ ions and higher temperature, led to a surplus of NW seeds. This, in turn, prohibited the formation of NWs of appreciable length because under these conditions, initiation of the reaction is favored rather than the propagation.

Based on these outcomes, the parameter space was tuned further to obtain a product of improved microstructure. The first results when EDA amount was increased to 0.3 mL at high pH were unsuccessful as the obtained NWs were merged and had rough surface (S20, Fig. 4). However, it was found that when the temperature of the process was additionally increased to 70 °C, such matrix-like formation did not occur, and the vast majority of the product contained straight NWs (S21, Fig. 4). Increased temperature disfavored deposition of present at higher concentration EDA at the ends of the NWs. When that surface was uncovered because of the delivery of an appropriate amount of thermal energy, the growth in the longitudinal direction was unlocked.

Finally, to optimize all the previously mentioned parameters, the effects of the prolonged time, elevated temperature, and increased amount of EDA were studied simultaneously. Firstly, experiment S18 with 0.2 mL of EDA at 70 °C for 60 min resulted in the formation of NWs of uniform diameter distribution, relatively good purity, but rather a short length (S18, Fig. 4). This was alleviated by increasing the amount of EDA up to 0.3 mL. Then, the length of these nanostructures was considerably increased without undesired growth in the radial directions (S22, Fig. 4). That indicated that under S18 conditions, the relative amount of reducing agent was too low in relation to other components necessary for the synthesis of NWs.

### Chemical composition of the synthesized NWs

**Figure 5:**
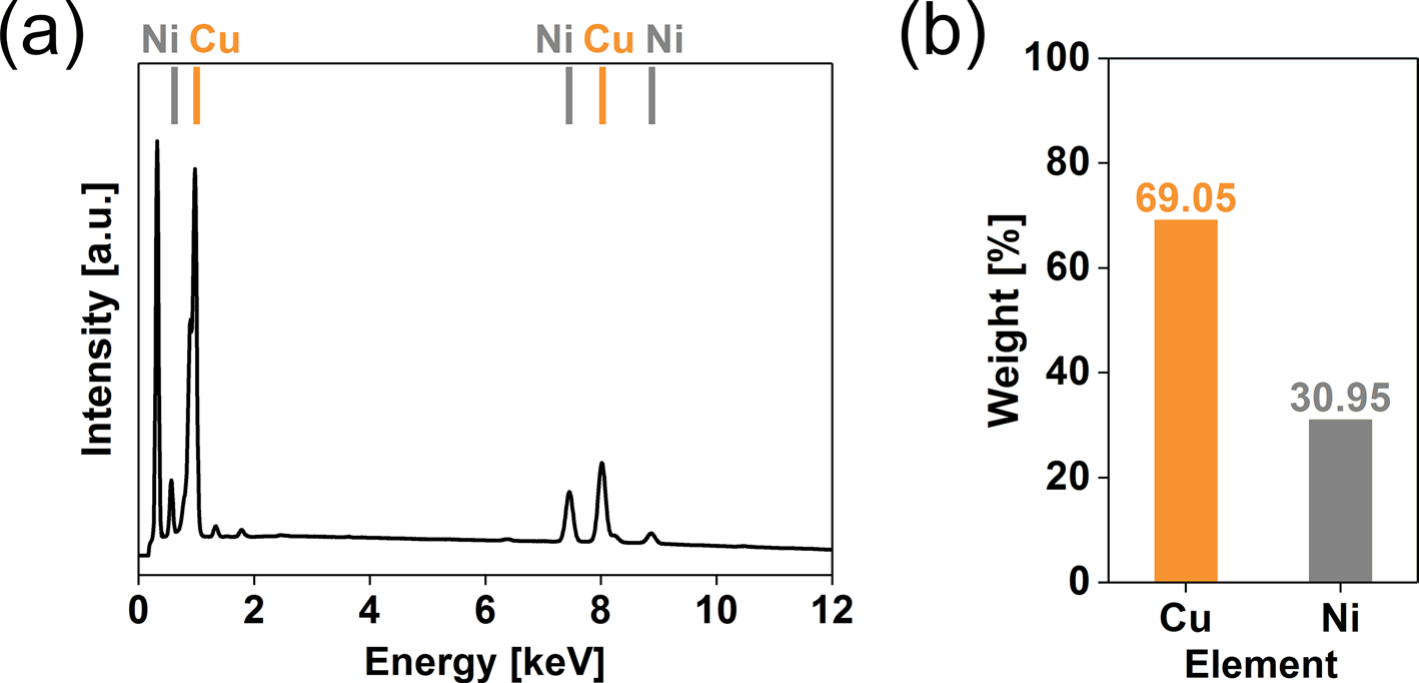
(**a**) EDX spectrum and (**b**) element composition diagram for Sample 15.

CuNi NWs of the best microstructure (S15, Fig. 4) were selected for the characterization of chemical composition (Fig. 5).

The EDX spectrum indicated that the weight ratio of copper to nickel was 2.23:1, which was higher than the ratio of these elements in the precursor solution (1.62:1). That was proof that precipitation of copper is more favorable than the deposition of nickel under these conditions. It is important to note that the purity of the produced NWs is exceptionally high as virtually no other elements were observed in the spectrum, regardless of the wide variety of the species present in the precursor solution such as As, Sb, Bi, Fe, and Al (Table 1).

**Figure 6:**
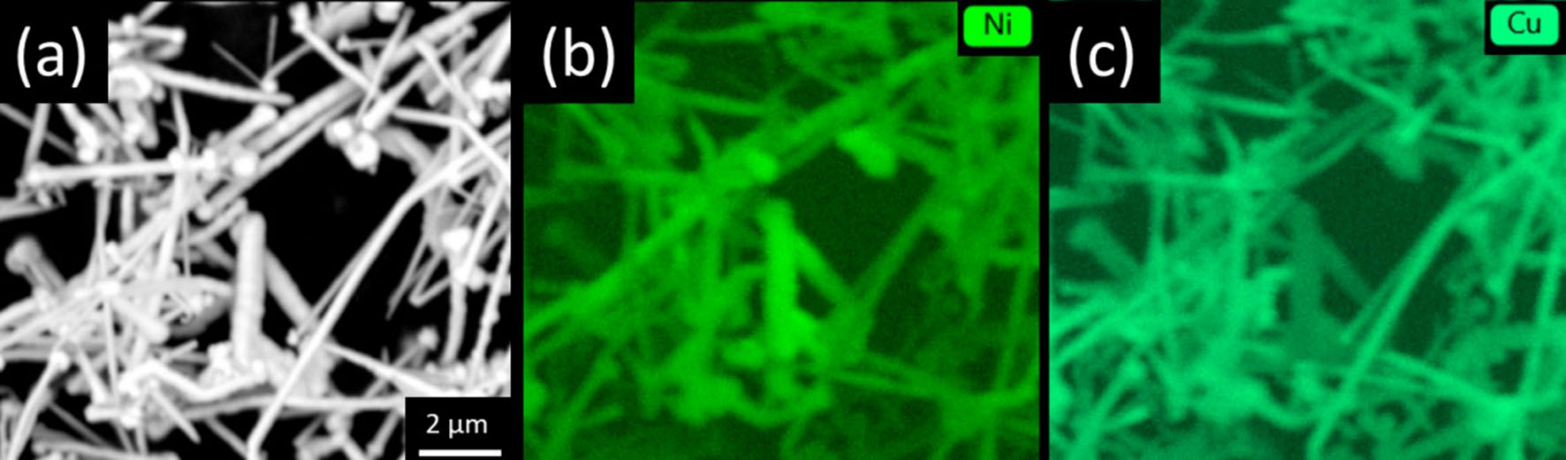
(**a**) SEM micrograph of Sample 15 with EDX spectra directed towards detection of (**b**) Ni and (**c**) Cu atoms.

To conclude, our results strongly suggest that the mechanistic aspect of the synthesis procedure is more complex in the case of complicated precursor mixtures (industrial process solutions) containing various species. EDX mapping showed that Cu and Ni atoms are deposited stochastically throughout the material (Fig. 6). No core–shell or any other specific configurations were observed, so these elements are blended to an appropriate degree. Nevertheless, some areas are richer in Cu or Ni. It cannot be excluded that more vigorous mixing at the molecular level should be employed to minimize this effect. Ultrasonication during synthesis could be employed to alleviate this effect, which we intend to study in the future. The properties of the produced NWs will also be a topic for further investigations.

## Conclusions

In summary, we have presented an inexpensive and quick way to produce composite CuNi NWs from complex industrial process solution in less than one hour. For the synthesis to be most successful, crucial parameters like capping agent concentration, pH, and temperature have to be carefully optimized to obtain straight and uniform NWs with small diameters rather than nanorods or nanoparticles. Then, the reaction time should be long enough (ca. 60 min in the present study) to support the anisotropic growth. The combination of 0.06 M Cu^2+^, 10 M NaOH, 0.3 mL EDA, 0.1 mL hydrazine, 70 °C and reaction time of 1 h (Sample 15) leads to NWs with the best morphology. First of all, the concentration of precursor ions is essential as its excessive amount favors radial growth. Therefore, solutions rich in metal ions may require higher than usual amounts of capping and reducing agents to promote anisotropic growth. Furthermore, we found that the content of these two reactants is crucial as well. The surplus of either of them gives rise to the generation of spherical nanoparticles. In the former case, too much capping agent encapsulates the nascent NW, metal ions cannot be added to elongate it, and hence its growth is terminated prematurely. In the former case, an oversupply of reducing agent creates too many seeds for NW growth, and so there is not enough precursor to sustain the synthesis. Lastly, we noticed that it is beneficial to prolong the synthesis time from 10 min to 1 h because after a short reaction time, the majority of the precursor remains unreacted. Besides that, it is worth noting that the presented methodology leads to NWs composed of exclusively Ni and Cu despite the use of multi-metal feed. Such materials have only been produced so far from synthetic solutions of high purity and less complicated nature. Optimization of the synthesis conditions and application of the magnetic field holds the key to gain more control over the NW growth and purification, respectively.

Moreover, we believe that this type of composite 1D nanostructures may be attractive to the R&D community. In this case, Ni provides magnetic properties, while Cu improves the electrical conductivity. The possibility of obtaining such bi-metallic or even multi-metallic materials can contribute to the development of materials with characteristics tailored for a particular application. These nanoarchitectures conceptually resemble co-polymers, which have gained significant attention and are currently present in many parts of modern life. More research is needed to understand the growth of composite nanostructures from complex solutions to exploit this concept to the fullest extent.

Last but not least, we should underline the ecological aspect of the proposed methodology. Increasing regulations due to environmental concerns put more and more focus on the development of new strategies that would align with the spirit of the circular economy. It is evident that we have to redesign our ways of processing by closing the open loops to make civilization more sustainable. We believe that by showing a tactic, which is very powerful in the recycling of heavy metal problematic waste and transforming it into a high added value product, we validated that this is indeed possible.
